# Molecular biomarkers associated with ATLL and HAM progression in HTLV-1 infection: a systematic review

**DOI:** 10.1590/S1678-9946202668030

**Published:** 2026-05-18

**Authors:** Theo Leite, Marcos Eduardo Souza Abreu, Alex Ap. Rosini Silva, Rubens de Assis Santos Sebastião, Lucas Araujo Romão, Jorge Casseb, Tatiane Assone, Fabio Eudes Leal, Sheila de Oliveira Garcia Mateos

**Affiliations:** 1 Universidade Municipal de São Caetano do Sul, Núcleo de Inovação em Saúde, São Caetano do Sul, São Paulo, Brazil; 2 Universidade de São Paulo, Faculdade de Medicina, Departamento de Dermatologia, São Paulo, São Paulo, Brazil; 3 Universidade de São Paulo, Faculdade de Medicina, Departamento de Medicina Legal, Bioética, Medicina do Trabalho e Medicina Física e Reabilitação, São Paulo, São Paulo, Brazil

**Keywords:** HTLV-1, Surrogate markers, Myelopathy associated disease (HAM, Adult T-cell leukemia/lymphoma (ATLL, Disease progression

## Abstract

Human T-lymphotropic virus type 1 (HTLV-1) infects approximately five to 10 million individuals worldwide, although only a minority develop severe outcomes such as adult T-cell leukemia/lymphoma (ATLL) or HTLV-1-associated myelopathy (HAM). Proviral load (PVL), while widely used, shows limited sensitivity and specificity, reinforcing the need for complementary biomarkers. Omics-based approaches have emerged as promising tools to improve risk prediction. We conducted a systematic review following PRISMA guidelines, searching PubMed, Web of Science, Virtual Health Library (BVS), and CAPES Periodicals databases for studies published between May 2020 and May 2025. Eligible studies included original observational designs investigating genomic, proteomic, and metabolic biomarkers associated with progression to ATLL or HAM. Methodological quality was assessed using tools from the National Heart, Lung, and Blood Institute (NHLBI). In total, 35 studies met the inclusion criteria, most conducted in Brazil, Japan, and Iran. A total of 67 biomarkers were identified: 37 genomic, 27 proteomic, and three metabolic with potential clinical applications in risk stratification, prognosis, and therapeutic monitoring of HTLV-1 infection. PVL remained the most frequently investigated marker but lacked predictive power in isolation. Additional candidates with strong potential included IFN-γ, CXCL10, Neopterin, AnxA1, and sTNFR2. This review highlights the potential of integrated multiparametric panels—combining PVL with omics-derived biomarkers—as a promising strategy to improve risk stratification, prognosis, and therapeutic monitoring in people living with HTLV-1 (PLHTLV-1). However, further longitudinal and clinically validated studies are needed to confirm their applicability and support their translation into early intervention strategies, particularly during the asymptomatic phase.

## INTRODUTION

Human T-lymphotropic virus type 1 (HTLV-1) was the first human retrovirus to be isolated and identified^1^, and it is estimated that there are between 5 and 10 million people living with HTLV-1 (PLHTLV-1) worldwide^2,3^. However, only about 3–5% of carriers develop severe associated complications, such as adult T-cell leukemia/lymphoma (ATLL) or HTLV-1-associated myelopathy (HAM), which poses challenges for early diagnosis and clinical management^4,5^.

HTLV-1 is primarily transmitted via sexual contact, blood transfusion, and breastfeeding, with higher prevalence in endemic regions such as southwestern Japan, sub-Saharan Africa, South America, the Caribbean, Austro-Melanesia, and specific foci in the Middle East^6^. HTLV-1 predominantly infects CD4+ T lymphocytes, promoting cell-to-cell transmission and clonal expansion of infected cells^7,8^. Diagnosis of infection is generally performed using serological assays such as enzyme-linked immunosorbent assay (ELISA), followed by western blot (WB) or real-time quantitative polymerase chain reaction (qPCR) to confirm and quantify the proviral load (PVL)^7,9^, which is widely used to infer the risk of clinical progression. However, PVL alone cannot predict disease development. Moreover, its utility is limited due to significant overlap between symptomatic and asymptomatic patients^10,11^.

Considering that currently available biomarkers still lack sufficient specificity and sensitivity to reliably identify individuals at high risk, particularly during the preclinical phase, there is growing interest in identifying additional markers that may improve diagnostic accuracy^12^. Omics sciences have emerged as approaches that enable the exploration of molecular mechanisms underlying HTLV-1 pathogenesis and its associated diseases. Potential biomarkers identified using these approaches may contribute to a better understanding of inflammatory, immunological, and metabolic processes associated with infection progression^13^. Thus, this systematic review aims to identify, categorize, and analyze the biomarkers reported in recent literature related to progression to ATLL and HAM, as well as to highlight existing gaps and potential strategies that may be applied in the future to address them.

## MATERIALS AND METHODS

This systematic review formulated its research question based on the PICO framework. The population (P) comprised individuals living with HTLV-1 infection across different clinical conditions, including ATLL, HAM, and asymptomatic carriers. The intervention/exposure (I) involved the assessment of genomic, proteomic, and metabolic biomarkers. The comparison (C) considered the use of PVL alone or conventional single-biomarker approaches. The outcomes (O) focused on improved risk stratification for disease progression and potential applications in disease monitoring.

Based on this framework, the guiding research question was: “Which biomarkers have been described in recent literature as being associated with progression from HTLV-1 infection to ATLL or HAM, compared with the use of PVL alone, and how do these biomarkers perform in risk stratification and disease monitoring?”

### Search strategies

The literature search was conducted up to May 2025 in the PubMed, Web of Science, Virtual Health Library (BVS), and CAPES Journals databases. The search period was restricted to the past five years to ensure that the review focused on the most recent and relevant evidence regarding biomarkers of interest. MeSH-based descriptors and related terms were used, combined with the Boolean operators ‘AND’ and ‘OR,’ and including synonyms to broaden coverage. The search strategy was as follows: ((“HTLV-1” OR “Human T-lymphotropic virus 1”) AND (“biomarkers” OR “markers” OR “genomics” OR “proteomics” OR “metabolomics” OR “omics”) AND (“TSP/HAM” OR “tropical spastic paraparesis” OR “ATLL” OR “adult T-cell leukemia” OR “ATL”)). No specific search for grey literature (e.g., theses or conference proceedings) was conducted to prioritize peer-reviewed studies, with preference given to open-access articles and full-text publications to facilitate analysis.

### Eligibility criteria

The Preferred Reporting Items for Systematic Reviews and Meta-Analyses (PRISMA) guidelines^14^ were applied to assist in article selection. Original studies published in English with full-text availability were included if they addressed biomarkers associated with progression to ATLL or HAM in PLHTLV-1. Studies were excluded if they used animal models, employed exclusively *in silico* approaches, involved pediatric populations, relied exclusively on publicly available datasets (e.g., KEGG, GEO, TCGA), or did not present original data. Additional exclusion criteria included studies addressing only serological or population genetics analyses, coinfections (such as HIV or hepatitis viruses), or those that did not compare at least two clinical groups (e.g., asymptomatic, HAM, ATLL, or infected symptomatic). Publications without original data (e.g., case reports, letters to the editor, comments, and reviews), duplicates, and studies that did not focus on molecular biomarkers or lacked sufficient information for qualitative synthesis were also excluded.

### Methodological quality assessment

Data analysis was conducted narratively, with qualitative synthesis of the findings, due to the heterogeneity of study designs and methodological approaches. Biomarkers were categorized according to their nature (genomic, proteomic, and metabolic) to facilitate interpretation. Study quality was assessed using tools from the National Heart, Lung, and Blood Institute (NHLBI, National Institutes of Health, USA)^15^. For case-control studies, the Quality Assessment of Case-Control Studies tool was used, classifying studies as good (> 8 criteria met, without critical “no” responses in items 1, 2, 4, 5, 6, 9, 10), fair (7–8 criteria), or “poor” (< 7 criteria). For cohort and longitudinal studies, the Quality Assessment Tool for Observational Cohort and Cross-Sectional Studies was applied, with studies classified as good (> 9 criteria, without critical “no” responses in items 1, 2, 4, 9, 11), fair (7–9 criteria), or poor (< 7 criteria). For cross-sectional studies, an adaptation of the latter tool was used, excluding items 8, 10, and 13. Studies were classified as good (> 7 criteria, without critical “no” responses in items 1, 2, 4, 9, 11), fair (6–7 criteria), or poor (< 6 criteria).

### Data extraction

Following inclusion and exclusion criteria, two researchers (TL and LAR) independently selected the studies and applied the quality assessment tools. Any discrepancies were assessed by a third reviewer (SOGM) and resolved by consensus. Data were extracted on authorship, year of publication, study design, country of origin, analyzed biomarker, laboratory techniques employed, and associated clinical outcomes, which were organized into tables to facilitate qualitative and comparative analysis.

## RESULTS

### Record selection

**Figure 1:**
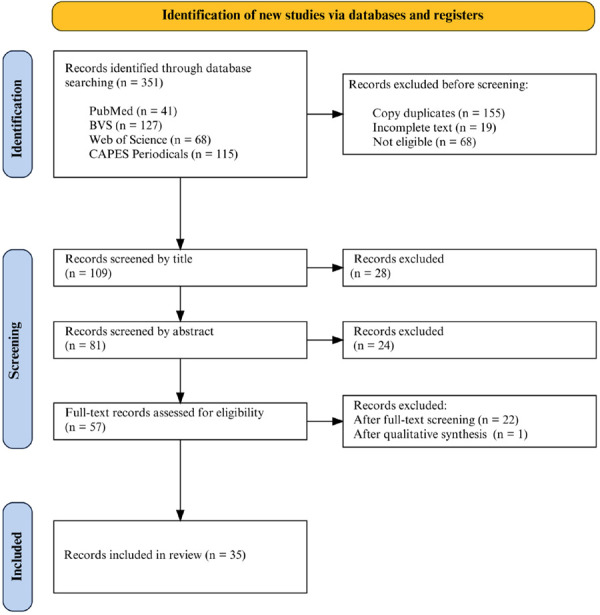
Flowchart for the identification and selection of articles.

Initially, 351 studies were identified across the databases (Figure 1). After removing duplicates, 109 potentially relevant studies remained. During screening, articles were excluded based on title and abstract, leaving 57 studies for full-text review and qualitative analysis, ultimately resulting in 35 articles that met the criteria and were included in the review.

### Study characteristics

The included studies comprised cross-sectional (n = 20), cohort (n = 9), case-control (n = 3), and longitudinal (n = 3) studies, conducted mainly in Brazil (n = 11), Japan (n = 11), Iran (n = 10), the United Kingdom (n = 3), and Argentina (n = 1). Population sizes ranged from 18 to 1,652 participants. Different samples—plasma, serum, peripheral blood mononuclear cells (PBMCs), cerebrospinal fluid (CSF), and biopsy tissue—were analyzed using molecular techniques. Genomic biomarkers were the most frequently investigated, followed by proteomic and metabolic markers. Recurring methodologies included ELISA, qPCR, enzyme-linked immunospot (ELISPot), flow cytometry, cytometric bead array (CBA), single-molecule array (SIMOA), and next-generation sequencing/RNA sequencing (NGS/RNA-seq), with additional complementary techniques. No biomarker demonstrated sufficiently high sensitivity and specificity for use in isolation. In total, 67 potential biomarkers were identified, including 37 genomic, 27 proteomic, and three metabolic, as shown in Table 1. More studies focused on HAM cases (n = 23) than on ATLL (n = 15), with only three studies including both conditions. For HAM, the most frequently used samples were plasma (n = 8), serum (n = 7), CSF (n = 6), and PBMCs (n = 12). For ATLL, plasma (n = 6), serum (n = 2), PBMCs (n = 5), peripheral blood (n = 3), and biopsy (n = 1) predominated, with some studies using more than one matrix. Seven studies declared conflicts of interest, involving funding from pharmaceutical companies, participation in advisory boards, affiliations with biotechnology firms, and patent registrations related to the biomarkers, while the remaining 28 studies reported no conflicts.

**Table 1:** Markers associated with ATLL and HAM progression in HTLV-1-infected individuals

Article	Country	Marker(s)	Category	HTLV Condition	Sample Type	Technique	Study Population (N)	Main Findings
Apoliano *et al*.^40^	Brazil	IFN-γ	Proteomic	HAM	PBMC	ELISPOT	89	Spontaneous IFN-γ three to four times higher in HAM
Assone *et al*.^16^	Brazil	GlycA	Metabolic	HAM	Plasma	H-NMR; Cytometric Bead Array	110	GlycA ~ IL-6 in Carriers; IFN-γ and IL-17A ↑ in HAM; IL-17A + PVL predict progression (64.1%)
		IL-6	Proteomic					
		IFN-γ						
		IL-17A						
Assone *et al*.^17^	Brazil	IL-10	Proteomic	HAM	Plasma	Cytometric Bead Array; Luminex Assay	110	IL-10 ↓ before PVL ↑
Benencio *et al*.^49^	Argentina	HLA-A*02 HLA-B*35 HLA-C*07	Genomic	ATLL; HAM	PBMC	Sequence Based Typing	66	HLA-A*02 → protection from ATLL; HLA-B*35 → susceptibility to HAM; HLA-C*07 → susceptibility to ATLL
Silva *et al*.^33^	Brazil/Japan	Neopterin	Metabolic	HAM	CSF	ELISA	75	CXCL-10 and Neopterin ↑ in HAM/
		CXCL10	Proteomic					
Freitas *et al*.^34^	Brazil	Neopterin	Metabolic	HAM	CSF; Serum	Multiplex Bead-Based Immunoassay	42	Fractalkine ↑ with low inflammation; BDNF ↑; TGF-β1 ↓; IL-18 ↑ with inflammation Neopterin and CXCL10 = markers of neuroinflammation
		CX3CL1 (Fractalkine)	Proteomic					
		BDNF						
		TGF-B1						
		IL-18						
		CXCL10						
Forghani-Ramandi^29^	Iran	ADCY1 ADCY3	Genomic	ATLL	PBMC	qPCR	20	ADCY1 and ADCY3 genes ↑ in ATLL
Gomes *et al*.^45^	Brazil	CHIT-1	Proteomic	HAM	CSF; Serum	Quantitative label-free proteomics LC-MS/MS; ELISA	43	V-CAM1 and CHIT-1 ↑ in HAM
		V-CAM1						
Ghobadi *et al*.^52^	Iran	FZD6 THBS4 SIRT1 CPNE3 miR-451a miR-142-3p	Genomic	ATLL	Peripheral Blood	qPCR	18	FZD6, THBS4, and miR-451a ↑ SIRT1, CPNE1, and miR-142-3p ↓
Guerrero *et al*.^21^	Japan	sTNFR2	Proteomic	ATLL	Plasma; PBMC	SOMAscan Assay; ELISA; Flow Cytometry; Immunostaining	102	sTNFR2 ↑ (~10x) in acute ATLL.
Harding *et al*.^10^	United Kingdom	T-cell activation markers (CD4/CD25, CD4/HLA-DR, CD8/CD25, CD8/HLA-DR)	Proteomic	HAM	Plasma	Flow Cytometry; SIMOA	216	PVL, T-cell activation markers (CD4/CD25, CD4/HLA-DR, CD8/CD25, CD8/HLA-DR), β2-microglobulin and Nf-L ↑ in HAM.
		β2-microglobulin						
		Nf-L						
Joris *et al*.^27^	United Kingdom	cfDNA	Genomic	ATLL; HAM	Plasma	qPCR	76	Plasma cfDNA HTLV-1 PVL detected in >85% of carriers; correlates with PBMC gDNA PVL in AC, HAM, and ATLL.
Katsuya *et al*.^19^	Japan	cfDNA	Genomic	ATLL	Plasma	ddPCR	67	cfDNA ↑ in aggressive ATLL, even with low PVL.
Kato *et al*.^23^	Japan	sTNFR2	Proteomic	ATLL	Plasma	ELISA	258	sTNFR-2 ↑ in acute ATLL.
Manzarinejad *et al*.^46^	Iran	PTX3	Proteomic	HAM	Plasma; Serum	ELISA	90	PTX3 ↑ in HAM.
Nakahata *et al*.^24^	Japan	sCADM1	Proteomic	ATLL	Plasma; Serum	AlphaLISA	364	sCADM1 ↑ in ATLL; decreases after treatment; normal in HAM
Nascimento *et al*.^51^	Brazil	miR-451a	Genomic	ATLL	PBMC	SmallRNA Sequencing	23	Five dysregulated sRNAs identified.
		miR-155						
		miR-146a						
		miR-150						
		miR-223						
Mozhgani *et al*.^32^	Iran	RhoA PKRACB	Genomic	ATLL	PBMC	qPCR	24	RhoA and PKRACB ↓ in ATLL.
Penova *et al*.^48^	Japan	DRB1-GB-7-Leu	Genomic	HAM	PBMC	GWA Study	1652	HLA-DRB1-GB-7-Leu: 9x ↑ risk for HAM.
Prates *et al*.^41^	Brazil	IFN-γ	Proteomic	HAM	PBMC	ELISPOT	328	IFN-γ ↑ in HAM and Intermmediate Syndrome; greater specifity for progression.
Rosadas *et al*.^35^	United Kingdom	Nf-L	Proteomic	HAM	CSF; Plasma	ELISA; SIMOA	49	Nf-L ↑ in CSF correlated with inflammation and disease duration; plasma levels also elevated. Positively correlates with CXCL10 and Neopterin.
		CXCL10						
		Neopterin	Metabolic					
Saeidi *et al*.^47^	Iran	XCL1	Proteomic	HAM	Serum	ELISA	179	XCL1 ↑ in HAM
Saffari *et al.* ^5^	Iran	Tax	Proteomic	HAM	PBMC	qPCR	56	Tax and IRF-1 expression ↑ (Tax ~130x) in HAM; HBZ shows no difference.
		IRF-1						
Santana *et al*.^20^	Brazil	AnxA1	Proteomic	HAM	Plasma	qPCR	104	AnxA1 ↑ in Carriers; PVL ↑ in HAM; combination = high sensitivity/specificity.
Shadabi *et al*.^30^	Iran	EVI1 MPK PTPRR JNK	Genomic	ATLL	Peripheral Blood	qPCR	18	EVI1, MKP1, PTPRR and MAPK8 (JNK) ↑ in ATLL vs healthy controls.
Shayeghpour*et al*.^31^	Iran	KMT2D	Genomic	ATLL	PBMC	qPCR	20	KMT2D ↓ in ATLL
Soltani *et al*.^44^	Iran	CCL3	Genomic	ATLL	Peripheral Blood	qPCR	30	Gene expression (mRNA) of CCL3, CCL4, CXCL8, and IL-17A ↑ in ATLL. (CCL3 > Carriers; CCL4/CXCL8 > controls).
		CCL4						
		CXCL8						
		IL-17A						
Souza *et al*.^22^	Brazil	Neopterin	Metabolic	HAM	CSF; Serum	ELISA	43	Neopterin and CXCL10 correlate with progression and axonal damage; CXCL10 ↑ 7x in CSF of HAM.
		CXCL10	Proteomic					
Souza *et al*.^18^	Brazil	hsa-miR-29b-3p	Genomic	HAM	PBMC	Small RNA sequencing qRT-PCR (for validation)	23	7 miRNAs ↓ in HAM, correlated to PVL
		hsa-let-7f-5p						
		hsa-miR-32-5p						
		hsa-miR-192-5p						
		hsa-miR-141-3p						
		hsa-miR-342-3p						
		hsa-miR-140-5p						
Tanaka *et al*.^50^	Japan	HLA-A*24	Genomic	HAM	PBMC	PCR-SSP	307	HLA-A*24 frequency ↑ in HAM vs ASY (72.4% versus 58.7%); HLA-A*24⁺ patients show ↓ PVL vs HLA-A*24⁻
Takenouchi *et al*.^25^	Japan	CADM1 (TSCL1)	Genomic	HAM	PBMC	qPCR; Flow Cytometry	30	CADM1 ↑ in HAM with severe symptoms
Yaghoubi *et al*.^7^	Iran	Nitric Oxide (NO)	Metabolic	HAM	Serum	Colorimetric Commercial Assay	100	MDA and NO ↑ in HAM (↑ Oxidative Stress); CAT, SOD, and GPX ↓
		Malondialdehyde (MDA)						
		Antioxidant enzymes (CAT, SOD e GPx)	Proteomic					
Yamada *et al*.^26^	Japan	Anti-Gag p19 and p24	Proteomic	ATLL; HAM	Plasma; Serum	LIPS Assay; NGS; ELISPOT	435	Anti-Tax/Env ↓ and Anti-Gag ↑ in ATLL. Anti-Gag/Tax ↑ in HAM
		Anti-Tax						
		Anti-Env						
Yamauchi *et al*.^36^	Japan	CXCL10	Proteomic	HAM	CSF	Cytometric Bead Array; High-Performance Liquid Chromatography (HPLC)	30	CXCL10/Neopterin ↑ in HAM → functional decline
		Neopterin	Metabolic					
Yanagida *et al*.^28^	Japan	SIRPα	Proteomic	ATLL	Tissue	Tissue Microarray	73	SIRPα+ associated with longer survival (28 vs. 16 months)

Potential biomarkers associated with the progression of adult T-cell leukemia/lymphoma (ATLL) and HTLV-1-associated myelopath (HAM) in HTLV-1-infected individuals, based on 35 selected studies. Biomarkers are categorized by their nature and related HTLV-1 condition, with details on type of sample, techniques employed, study population (N), and main findings.

### Methodological quality assessment

Regarding methodological quality, 20 were rated as good and 15 as fair, no low-quality studies were included (Table 2). All studies demonstrated high quality in the title, abstract, introduction, and description of participant characteristics, providing clear objectives and appropriate context. Studies rated as good were notable for well-defined populations, consistent analytical methods, and clear eligibility criteria. In contrast, studies rated as fair showed limitations, such as lack of sample size justification or absence of reporting on participation rates, partially compromising robustness. Despite methodological heterogeneity of study designs and variability in laboratory techniques, the exclusion of low-quality studies and adherence to most critical criteria ensure reliability of the findings.

**Table 2:** Methodological quality assessment of included studies

Author	Country	Quality	1	2	3	4	5	6	7	8	9	10	11	12	13	14
Case-control studies																
Yaghoubi *et al*.^7^	Iran	Fair	Y	Y	N	Y	Y	Y	NR	Y	Y	Y	NR	N	–	–
Guerrero *et al.* ^21^	Japan	Good	Y	Y	N	Y	Y	Y	Y	Y	Y	Y	NR	N	–	–
Assone *et al.* ^16^	Brazil	Good	Y	Y	N	Y	Y	Y	NR	Y	Y	Y	Y	Y	–	–
Benencio *et al*.^49^	Argentina	Fair	Y	Y	N	Y	Y	Y	NR	Y	Y	Y	NR	N	–	–
Shayeghpour *et al*.^31^	Iran	Fair	Y	Y	N	Y	Y	Y	NR	Y	Y	Y	NR	N	–	–
Tanaka *et al.* ^50^	Japan	Fair	Y	Y	N	Y	Y	Y	NR	Y	Y	Y	NR	N	–	–
Forghani-Ramandi *et al*.^29^	Iran	Fair	Y	Y	N	Y	Y	Y	NR	Y	Y	Y	NR	N	–	–
Cohort studies																
Nakahata *et al*.^24^	Japan	Good	Y	Y	N	Y	N	Y	Y	Y	Y	Y	Y	NR	NR	Y
Rosadas *et al.* ^35^	UK	Good	Y	Y	N	Y	N	Y	Y	Y	Y	Y	Y	NR	NR	Y
Kato *et al.* ^23^	Japan	Good	Y	Y	Y	Y	Y	Y	Y	Y	Y	N	Y	NR	Y	Y
Yanagida *et al.* ^28^	Japan	Fair	Y	Y	N	Y	N	Y	Y	Y	Y	N	Y	NR	NR	Y
Yamauchi *et al.* ^36^	Japan	Good	Y	Y	N	Y	N	Y	Y	Y	Y	Y	Y	NR	NR	Y
Longitudinal studies																
Assone *et al.* ^17^	Brazil	Good	Y	Y	Y	Y	Y	Y	Y	Y	Y	Y	Y	NR	NR	Y
Freitas *et al.* ^34^	Brazil	Good	Y	Y	Y	Y	N	Y	Y	Y	Y	Y	Y	NR	Y	Y
Harding *et al.* ^10^	UK	Good	Y	Y	N	Y	N	Y	Y	Y	Y	Y	Y	NR	Y	Y
Cross-sectional studies																
Souza *et al.* ^22^	Brazil	Good	Y	Y	Y	Y	N	Y	NA	Y	Y	NA	Y	NR	NA	Y
Penova *et al.* ^48^	Japan	Good	Y	Y	NR	Y	N	Y	NA	Y	Y	NA	Y	NR	NA	Y
Nascimento *et al.* ^51^	Brazil	Fair	Y	Y	NR	Y	N	Y	NA	Y	Y	NA	Y	NR	NA	N
Yamada *et al.* ^26^	Japan	Good	Y	Y	NR	Y	N	Y	NA	Y	Y	NA	Y	NR	NA	Y
Gomes *et al.* ^45^	Brazil	Fair	Y	Y	NR	Y	N	N	NA	Y	Y	NA	Y	NR	NA	N
Saffari *et al.* ^5^	Iran	Fair	Y	Y	NR	Y	N	Y	NA	Y	Y	NA	Y	NR	NA	N
Silva *et al.* ^33^	Brazil/Japan	Good	Y	Y	Y	Y	N	Y	NA	Y	Y	NA	Y	NR	NA	Y
Takenouchi *et al.* ^25^	Japan	Fair	Y	Y	NR	Y	N	Y	NA	Y	Y	NA	Y	NR	NA	N
Soltani *et al.* ^44^	Iran	Good	Y	Y	NR	Y	Y	Y	NA	Y	Y	NA	Y	NR	NA	Y
Katsuya *et al.* ^19^	Japan	Good	Y	Y	NR	Y	N	Y	NA	Y	Y	NA	Y	NR	NA	Y
Souza *et al.* ^18^	Brazil	Good	Y	Y	NR	Y	N	Y	NA	Y	Y	NA	Y	NR	NA	Y
Apoliano *et al.* ^40^	Brazil	Good	Y	Y	NR	Y	N	Y	NA	Y	Y	NA	Y	NR	NA	Y
Santana *et al.* ^20^	Brazil	Good	Y	Y	Y	Y	N	Y	NA	Y	Y	NA	Y	NR	NA	Y
Manzarinejad *et al.* ^46^	Iran	Fair	Y	Y	NR	Y	N	Y	NA	Y	Y	NA	Y	NR	NA	N
Prates *et al.* ^41^	Brazil	Good	Y	Y	Y	Y	N	Y	NA	Y	Y	NA	Y	NR	NA	Y
Shadabi *et al.* ^30^	Iran	Fair	Y	Y	NR	Y	N	Y	NA	Y	Y	NA	Y	NR	NA	N
Gbobadi *et al.* ^52^	Iran	Fair	Y	Y	NR	Y	N	Y	NA	Y	Y	NA	Y	NR	NA	N
Joris *et al.* ^27^	UK	Fair	Y	Y	NR	Y	Y	N	NA	Y	Y	NA	Y	NR	NA	N
Mozhgani *et al.* ^32^	Iran	Fair	Y	Y	NR	Y	N	N	NA	Y	Y	NA	Y	NR	NA	N
Saeidi *et al.* ^47^	Iran	Good	Y	Y	NR	Y	N	Y	NA	Y	Y	NA	Y	NR	NA	Y

Y = criterion met; N = criterion not met; NR = not reported; NA = not applicable; – = criterion not present in the original guideline.

## DISCUSSION

This review demonstrates that HAM is predominantly associated with chronic inflammation and neurodegeneration, whereas ATLL is mainly linked to tumor-related and immunomodulatory markers. Although many available studies remain exploratory and require validation in larger cohorts, several biomarkers hold strong diagnostic and prognostic potential. However, only three studies adopted a longitudinal design, while the majority were cross-sectional, limiting the assessment of slow and gradual disease progression.

Our analysis identified genomic, proteomic, and metabolic biomarkers associated with progression to ATLL and HAM in PLHTLV-1, highlighting the potential of omics sciences for identifying novel and specific biomarkers. Among markers already used in clinical practice, PVL, measured by qPCR, was frequently reported across several studies^5,10,16-20^. Consistently elevated in patients with clinical outcomes—particularly those with HAM and acute forms of ATLL—PVL supports its role as a risk indicator, with its predictive value further enhanced when combined with other biomarkers^5,10,16,19,21,22^.

In the context of ATLL, key biomarkers identified included sTNFR2 and sCADM1. Two studies reported plasma concentrations of sTNFR2 to be approximately tenfold higher in patients with acute disease compared to asymptomatic individuals. Elevated levels were also frequently detected in ATLL cells, increasing progressively with disease advancement. Both biomarkers showed significant decreases following treatment initiation, supporting their potential use as monitoring tools.

Furthermore, sTNFR2 showed strong correlations with sCD25, sOX40, IL-10, and PVL, and sCADM1 correlated with PVL and other clinical parameters. These findings suggest that simultaneous increases may signal progression to acute ATLL^21,23-25^. Similarly, alterations in the humoral immune response against HTLV-1 proteins have been identified in ATLL and HAM patients. Decreased levels of anti-Tax and anti-Env antibodies were observed in ATLL patients compared to HTLV-1 carriers and HAM patients, while anti-Gag p19 and p24 levels were significantly elevated in ATLL. Conversely, HAM patients exhibited elevated anti-Gag and anti-Tax antibodies^26^. Plasma levels of cell-free DNA (cfDNA) were also found to be elevated in HTLV-1-infected individuals, including asymptomatic carriers and patients with HAM, with the highest levels observed in patients with aggressive ATLL. Notably, the lymphoma subtype exhibited higher cfDNA levels even when PVL was low, suggesting that cfDNA may serve as a more sensitive marker of tumor burden than PBMC-derived PVL in this subgroup^19,27^. Finally, the expression of the signal regulatory protein alpha (SIRPα), identified with biopsy analysis, revealed a potential prognostic marker: patients negative for SIRPα had a median survival of 16 months after diagnosis, whereas SIRPα-positive patients reached a median survival of 28 months^28^.

Several genomic biomarkers have been proposed for ATLL. In this context, qPCR validation confirmed significant upregulation of ADCY1 and ADCY3 in patients with ATLL. These genes encode adenylyl cyclases, key enzymes of the cyclic adenosine monophosphate (cAMP) production pathway, which catalyze the conversion of ATP into cAMP following G protein-coupled receptor activation. cAMP is responsible for cell proliferation, regulation of apoptosis, and response to DNA damage^29^. Additionally, experimental validation demonstrated increased expression of MAPK8 (JNK) together with its negative regulators EVI1, MKP-1, and PTPRR, indicating dysregulated MAPK/JNK signaling pathway in ATLL and further supporting the presence of altered intracellular signaling networks contributing to leukemogenesis^30^.

Moreover, qPCR analysis demonstrated significant downregulation of KMT2D, suggesting that epigenetic deregulation may also contribute to ATLL pathogenesis. In the same study, although UBB and RPS15A were experimentally validated, the authors concluded that their reduced expression levels do not support a pathogenic or biomarker role in ATLL^31^. RhoA and PRKACB were also found to be downregulated in ATLL patients compared with asymptomatic individuals, suggesting loss of their tumor-suppressive activity in infected cells, which may contribute to increased tumorigenicity, cancer progression, and reduced apoptosis^32^. However, all these genomic findings are limited by small sample sizes, and further studies in larger cohorts are required for validation.

For HAM, the combination of CXCL10 and Neopterin stood out as consistent indicators of disease progression, being detected in CSF and associated with central nervous system (CNS) inflammatory activity, as reported in five studies^22,33-36^. HAM patients exhibited CXCL10 levels up to sevenfold higher than asymptomatic individuals or controls, particularly in cases of accelerated clinical progression^22,35^. Together with Neopterin, CXCL10 proved superior to neuronal injury markers^34^, such as phosphorylated heavy neurofilament (pNf-H)^22^ and light neurofilament (Nf-L)^35^, showing proportional increases with disease progression rate and serving as an indicator of clinical stage and degree of neurological impairment^22^. These findings reflect IFN-γ-mediated immune activation and persistent neuroinflammation in the CNS^34,37^. Supporting this scenario, Espíndola *et al*.^38^ demonstrated a higher frequency and persistence of IFN-γ and IL-10 expression in infected clones, complementing recent data showing that lower plasma levels of IL-10 predict increased PVL and clinical progression, suggesting a protective role for this interleukin^17^.

In this context, IFN-γ has been a central focus of investigation. As one of the most abundant pro-inflammatory cytokines in patients with HAM, it has been hypothesized that its excessive production contributes to chronic CNS inflammation, leading to neuronal and myelin sheath damage^39^. Three studies included in this review investigated IFN-γ^16,40,41^, and, as previously described in the literature, it was consistently found at significantly higher levels in untreated HAM patients, including during intermediate disease stages, compared to asymptomatic individuals^40,42^. Using the ELISPot technique, one study demonstrated that spontaneous IFN-γ production by PBMCs was three- to fourfold higher in HAM patients compared to asymptomatic individuals^40^, reinforcing the findings of other studies^16,41^. This IFN-γ hyperactivity not only confirms its involvement in the neuroinflammatory process but also revealed a positive correlation with PVL, making IFN-γ a promising biomarker for risk stratification in these patients^43^. Moreover, it was observed that the combination of IFN-γ and IL-17A—both elevated in untreated HAM patients—can achieve a predictive accuracy of up to 62.1% for clinical progression when combined with PVL^16^.

Interestingly, although IL-17A was initially associated with HAM, its gene expression levels (mRNA) were also elevated in ATLL patients, indicating that its immunopathological role is not limited to a single disease. Furthermore, significant differences in expression were observed between analyzed groups (p < 0.05)^44^. As IFN-γ can be quantified or its activity measured in both plasma and PBMCs using minimally invasive methods^16,40,41^, it stands out as a promising core marker for future studies. These studies could investigate additional markers, such as IL-17A itself^16^, which could be combined with this cytokine to enhance predictive capacity regarding infection progression.

Other proteomic biomarkers have also been identified as indicators of HAM activity and progression. Elevated expression of V-CAM and CHIT-1 proteins has been reported, with CHIT-1 notably associated with pro-inflammatory responses in other neurodegenerative disorders. CHIT-1, in particular, has been proposed as a candidate marker for clinical stage and progression type, particularly in rapidly evolving cases with poor prognosis^45^. Similarly, pentraxin 3 (PTX3) protein was found at significantly elevated levels in the serum and plasma of patients with HAM compared to asymptomatic carriers and healthy controls, with no significant differences observed between the other groups^46^. Another relevant biomarker was the chemokine XCL1, whose plasma levels were significantly elevated in patients with HAM. This finding may be associated with facilitation of autoreactive T cell migration into the CNS. Notably, XCL1 levels were markedly higher in HAM patients than in individuals with multiple sclerosis, suggesting greater specificity in the context of HTLV-1 infection^47^.

In addition to these findings, other biomarkers were evaluated in combination with PVL. One example is the viral protein Tax, whose expression was found to be approximately 130-fold higher in symptomatic patients. When combined with PVL, Tax improved differentiation between symptomatic and asymptomatic individuals and was also associated with increased IRF-1 expression^5^. Another promising biomarker, AnxA1, was found at elevated levels in asymptomatic PLHTLV-1. Combined analysis of AnxA1 with PVL showed high sensitivity and specificity, suggesting potential for early diagnosis and estimation of the risk of clinical progression to HAM^20^.

From the genomic perspective, relevant biomarkers have also been identified. A notable finding is the association between the HLA-DRB1-GB-7 allele and increased disease susceptibility, as demonstrated in a genome-wide association study (GWAS). Individuals homozygous for this allele had up to a ninefold higher risk of disease development^48^. Additionally, HLA class I alleles have been implicated in modulating clinical outcomes. The HLA-B*35 allele has been associated with increased susceptibility to HAM, whereas HLA-A*02 has demonstrated a protective effect against ATLL. Independent studies have also reported a protective role of HLA-A*02 in HAM. Furthermore, HLA-C*07 has been linked to ATLL progression^49^. The HLA-A*24 allele was also presented as a relevant susceptibility marker, being more frequent among HAM patients despite its association with lower HTLV-1 proviral load^50^.

Beyond HLA variants, microRNA profiling revealed seven miRNAs (hsa-miR-29b-3p, hsa-let-7f-5p hsa-miR-32-5p, hsa-miR-192-5p, hsa-miR-141-3p, hsa-miR-342-3p, and hsa-miR-140-5p) that were significantly downregulated in patients with HAM. These miRNAs were correlated with PVL and involved in the regulation of cellular senescence and modulation of inflammatory pathways, such as NF-κB and TGF-β^18^. These findings are consistent with another study that reported reduced TGF-β1 and increased BDNF levels in carriers and patients with HAM, reinforcing the hypothesis that dysfunction of anti-inflammatory pathways may contribute to disease progression^34^. Another study identified five dysregulated small RNAs (miR-451a, miR-155, miR-146a, miR-150, and miR-223) associated with key signaling pathways relevant to both HAM and ATLL, including TGF-β, p53, MAPK, PI3K, and Wnt pathways, which are critical regulators of proliferation, apoptosis, and inflammatory responses^51^. Further experimental validation in ATLL demonstrated significant upregulation of miR-451a and downregulation of miR-142-3p in ATLL patients compared to healthy controls. These miRNAs were shown to regulate genes involved in immune modulation, cell migration, angiogenesis, and oncogenic signaling pathways, reinforcing the role of disrupted miRNA-mediated regulatory networks as a shared molecular feature underlying HTLV-1-associated disease progression, including both HAM and ATLL^52^.

Biomarkers derived from metabolic profiling have also gained attention due to their clinical applicability, as they reflect systemic aspects of the inflammatory response. In addition to these, GlycA—a nuclear magnetic resonance-derived marker that reflects circulating glycosylated acute-phase proteins—has recently emerged as a serum indicator of chronic systemic inflammation^53^. In asymptomatic individuals, a positive correlation between IL-6 and GlycA levels has been observed, consistent with previous findings in uninfected populations. However, this correlation was not detected in patients with HAM, possibly due to the predominantly neuroinflammatory nature of the disease. The lack of association between GlycA and IL-6 may indicate a distinct inflammatory pattern in this group^16^.

Oxidative stress has also been associated with HTLV-1 infection, particularly in cases progressing to HAM. Antioxidant enzymes, including superoxide dismutase (SOD), catalase (CAT), and glutathione peroxidase (GPX), exhibit reduced activity in PLHTLV-1, whereas serum levels of major oxidative damage markers—such as malondialdehyde and nitric oxide—are elevated, especially in symptomatic individuals. This imbalance suggests a progressive increase in oxidative stress across different infection stages. These findings highlight a potential therapeutic window based on modulation of the redox system to prevent or delay clinical progression of the infection^7^. Moreover, these results align with previous reports of reduced total antioxidant capacity in PLHTLV-1^54^, as well as with similar patterns observed in other chronic viral infections, such as human immunodeficiency virus (HIV)^55^. HIV-1 studies have demonstrated that, during the viral latency phase, specific metabolic pathways exhibit reduced activity, raising the hypothesis that similar mechanisms may also occur in HTLV-1 infection^56^.

The consistent identification of multiple biomarkers in this systematic review emphasizes the possibility of developing an integrated panel combining proteomic, genomic, and metabolic markers. Such an approach would provide a strategic framework for mapping research gaps and priorities, thereby facilitating the identification of underexplored areas. Among the omics sciences analyzed in this study, metabolomics was the least investigated, with only three metabolic markers identified, all associated with HAM, and only two obtained using metabolomic methods (nuclear magnetic resonance and mass spectrometry)^16,36^.

Given its potential to unravel complex molecular mechanisms, metabolomics represents a valuable approach to elucidate how alterations in metabolic pathways directly influence HTLV-1 pathogenesis and clinical outcomes. Exploratory strategies, such as untargeted metabolomics, enable the simultaneous profiling of multiple metabolites within a single sample^57^, facilitating the identification of novel patterns and correlations among metabolic pathways, previously reported consistent biomarkers, and clinical data. Hence, metabolomics may serve as a valuable tool to address current gaps in understanding pathophysiological mechanisms underlying HTLV-1 infection.

Limitations of this review include the heterogeneity of study designs, which limits direct comparisons between findings, as well as the predominance of small sample sizes. Methodological quality, assessed using NHLBI tools, indicated that 15 studies were rated as fair, mainly due to the lack of sample size justification and insufficient information regarding assessor blinding, control of confounding variables, and loss to follow-up. Furthermore, compared to HAM, considerably fewer studies focused on ATLL. This discrepancy may be explained by the short survival of patients who progress to aggressive clinical forms of ATLL—approximately eight to 10 months for the acute, lymphoma, and unfavorable chronic subtypes, and two to four years for indolent forms, such as favorable chronic and smoldering subtypes. This limited time frame poses challenges for cohort establishment and biological sample collection^23,58^. Despite these limitations, the overall quality and relevance of this systematic review remain robust. The inclusion of high and moderate methodological quality, supported by rigorous assessment, combined with a detailed narrative analysis and adherence to PRISMA guidelines^14^, ensures reliability of the data. Gray literature was excluded and language was restricted to minimize bias and avoid the inclusion of unvalidated data. While gray sources may provide additional insights, their limited quality and reproducibility justified exclusion, with relevant references still used to mitigate this limitation.

Finally, considering the observed gaps, particularly regarding metabolic markers, future studies are recommended to prioritize longitudinal cohorts of asymptomatic individuals to identify predictive markers during the preclinical phase^59^. The integration of machine learning-based approaches for omics data analysis could enhance the identification of predictive patterns, as has already been demonstrated in hematologic malignancies^60^. International collaborations, particularly in endemic regions such as Brazil and Japan, as exemplified by Silva *et al*.^33^, are important strategies to overcome sample size limitations and reduce geographic bias. Ultimately, clinical validation of identified markers will require well-designed multicenter prospective trials.

## CONCLUSION

This systematic review, based on 35 studies of high and moderate methodological quality, compiled genomic, proteomic, and metabolic biomarkers with potential applications in risk stratification, prognosis, and therapeutic monitoring of progression to ATLL and HAM in PLHTLV-1. Although PVL remains the primary clinical marker, its limited specificity underscores the need for combination with other biomarkers such as IFN-γ, CXCL10, Neopterin, and sTNFR2. This review also reinforces that no single marker is sufficient; instead, multiparametric panels of correlated biomarkers are required. Metabolomics, although still underexplored, appears to be a promising approach to fill gaps in future investigations, enabling the discovery of novel markers with the understanding of metabolic pathways and infection pathogenesis, as well as their correlation with previously identified markers. The consistent findings across omics categories support the feasibility of developing integrated biomarker panels to enhance clinical decision-making. Additionally, longitudinal studies integrating omics approaches and artificial intelligence are needed to improve predictive accuracy, enable early intervention, and ultimately guide personalized clinical management in HTLV-1 infection, particularly during the asymptomatic phase.

## Data Availability

The complete anonymized dataset supporting the findings of this study is included within the article.
